# Impact of changing the measles vaccine vial size on Niger's vaccine supply chain: a computational model

**DOI:** 10.1186/1471-2458-11-425

**Published:** 2011-06-02

**Authors:** Tina-Marie Assi, Shawn T Brown, Ali Djibo, Bryan A Norman, Jayant Rajgopal, Joel S Welling, Sheng-I Chen, Rachel R Bailey, Souleymane Kone, Hailu Kenea, Diana L Connor, Angela R Wateska, Anirban Jana, Stephen R Wisniewski, Willem G Van Panhuis, Donald S Burke, Bruce Y Lee

**Affiliations:** 1 University of Pittsburgh, Pittsburgh, PA, USA; 2 Pittsburgh Supercomputing Centre, Pittsburgh, PA, USA; 3 Niger Ministry of Health, Niamey, Niger; 4 World Health Organization, Geneva, Switzerland

**Keywords:** Measles Vaccine, Vaccine Supply Chain, Niger

## Abstract

**Background:**

Many countries, such as Niger, are considering changing their vaccine vial size presentation and may want to evaluate the subsequent impact on their supply chains, the series of steps required to get vaccines from their manufacturers to patients. The measles vaccine is particularly important in Niger, a country prone to measles outbreaks.

**Methods:**

We developed a detailed discrete event simulation model of the vaccine supply chain representing every vaccine, storage location, refrigerator, freezer, and transport device (e.g., cold trucks, 4 × 4 trucks, and vaccine carriers) in the Niger Expanded Programme on Immunization (EPI). Experiments simulated the impact of replacing the 10-dose measles vial size with 5-dose, 2-dose and 1-dose vial sizes.

**Results:**

Switching from the 10-dose to the 5-dose, 2-dose and 1-dose vial sizes decreased the average availability of EPI vaccines for arriving patients from 83% to 82%, 81% and 78%, respectively for a 100% target population size. The switches also changed transport vehicle's utilization from a mean of 58% (range: 4-164%) to means of 59% (range: 4-164%), 62% (range: 4-175%), and 67% (range: 5-192%), respectively, between the regional and district stores, and from a mean of 160% (range: 83-300%) to means of 161% (range: 82-322%), 175% (range: 78-344%), and 198% (range: 88-402%), respectively, between the district to integrated health centres (IHC). The switch also changed district level storage utilization from a mean of 65% to means of 64%, 66% and 68% (range for all scenarios: 3-100%). Finally, accounting for vaccine administration, wastage, and disposal, replacing the 10-dose vial with the 5 or 1-dose vials would increase the cost per immunized patient from $0.47US to $0.71US and $1.26US, respectively.

**Conclusions:**

The switch from the 10-dose measles vaccines to smaller vial sizes could overwhelm the capacities of many storage facilities and transport vehicles as well as increase the cost per vaccinated child.

## Background

Many countries, such as Niger, are considering changing their vaccine vial size presentations, i.e. number of doses per vial. Single-dose vials reduce contamination risk, inaccurate dosing, and vaccine wastage, but increase space requirements, medical waste volume and costs per dose[1-4]. Countries changing vaccine vial size presentations in their World Health Organization (WHO) Expanded Programmes on Immunization (EPI) should evaluate the subsequent impact on their supply chains, the series of steps required to get vaccines from their manufacturers to patients. The measles vaccine is particularly important in Niger, a country prone to measles outbreaks[5,6]. In 2003, health officials in Niger reported 50,138 cases and 201 deaths from measles[5]. Providing vaccines to children, especially in rural areas, has been challenging[5,6].

The Vaccine Modelling Initiative (VMI), funded by the Bill and Melinda Gates Foundation, collaborated with the Ministry of Health and WHO in Niger and other partners, to develop a computational model of the national vaccine supply chain. We developed a model of the Niger vaccine supply chain representing the flow of all WHO EPI vaccines from manufacturers, to the central storage facility (Niamey), through each subsequent level of the supply chain, and finally to vaccine recipients at integrated health centres (IHCs) (i.e., service delivery level). Using this model, we simulated the replacement of multi-dose measles vaccines with vaccines in smaller vial sizes to determine the impacts on the supply chain.

## Methods

### General Framework

The Highly Extensible Resource for Modelling Supply Chains (HERMES), a custom-designed, dynamic, discrete event simulation model (DES), is written in the Python programming language, using features provided by the SimPy package[7]. This model explicitly simulates all processes, storage locations, administering locations, and storage equipment in the Niger vaccine supply chain.

### Niger Vaccine Supply Chain and Data Sources

**Figure 1:**
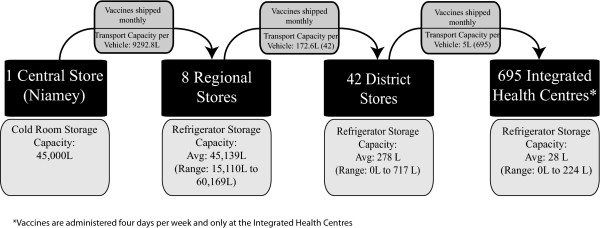
**Niger Vaccine Supply Chain Network**.

Figure 1 shows the four levels of the supply chain. Data to construct the models was collected from the WHO in Geneva, WHO in Niger, UNICEF, the Niger National Geographic Institute (NGI), the Niger Ministry of Health (MOH), the WHO EPI in Niger, and direct field observations. In 2009, the following data was collected in-country to begin model development: cold chain equipment inventory[8]; transportation resources; operating polices for shipments, storage, and aspects of vaccine administration; and patterns of patient arrival.

The number of vaccines administered at the IHCs is a function of the population demand. The population demand is estimated using district-level birth registry data from 2005 (557,381 total newborns across all districts) adjusted by an annual growth rate of 1.04% to 2010 (586,880 total newborns across all districts) to account for population growth[9]. Newborns are distributed among the 695 IHC sites, and in each of the immunization sessions each month. Each time a patient arrives for vaccination at an IHC, he or she receives the appropriate age-specified (0-11 month, and 12-24 month old children) vaccines, if they are available.

### Model Structure

We chose to utilize a DES model because the effects of changing the measles vaccine vial size may be subtle, complex, dynamic and not captured by less detailed representations. For example, changing vial size can affect the ability of a clinic to fulfil demand and the amount of open vial wastage, which affects a clinic's order sizes, effects that may propagate up the supply chain.

In HERMES, each vaccine vial is an entity complete with its own set of relevant characteristics such as vaccine antigen type, doses per vial, shelf-life, formulation, and packaged volume. Each Niger EPI vaccine vial [i.e., Bacille Calmette-Guérin tuberculosis (BCG), oral polio (OPV), measles, yellow fever (YF), tetanus toxoid (TT), and the diphtheria-tetanus-pertussis-haemophilus influenzae type B-hepatitis B (DTP-HepB-Hib) vaccines] is represented as an entity. Each vaccine vial entity begins at the manufacturer and is packaged into a shipping lot with other vial entities. From there, the entity gets delivered with the shipping lot to the central store where the entity is unloaded, repackaged into a smaller lot and stored in a cold room until it is scheduled for further shipment. When shipment time comes, depending on its destination, it will either be loaded into cold trucks destined for regional stores, or 4 × 4 trucks destined for district stores, where it will be unloaded, repackaged into a smaller lot and stored in a cold room, refrigerator or freezer. A district store will use its 4 × 4 truck to collect the entity in its lot whenever its inventory is depleted by downstream clinics. A clinic will then send its vaccine carrier to its district store to collect the entity in its lot when the clinic reaches its re-order point. At each storage location and in transport vehicle, an entity has a probability of breakage from mishandling. The entity also has a specified lifetime beyond which its doses expire.

Based on WHO recommendations, HERMES assumes that clinics and district stores re-order points are 25% of their maximum and that orders will account for a 25% buffer[10]. When cold space is limited, HERMES uses a balanced allocation scheme wherein complete vaccine regimens (i.e., all doses required for full immunization) are prioritized over single antigens, and a 'first-in-first-out' policy is used every time a vaccine is removed from a storage or transport device. Each simulation represents a one-year time horizon over which statistics on vaccination rates, stock-outs, vial expiration, and storage and transport utilization are accumulated.

Each refrigerator maintains a temperature of 2°C to 8°C and each freezer a temperature of -15°C to -25°C. Each vaccine's required temperature profile determines whether it will be stored in freezer or refrigerator compartments.

Current vaccine inventory in each cold room, freezer, refrigerator, or transport device (i.e., the number of vaccines currently stored in that device) is equal to the number of vaccines arriving that day plus the number of vaccines left over from the previous day minus the number of vaccines removed (to either be shipped or administered).The model does not allow the total vaccine inventory stored in a refrigerator, freezer, or cold room to ever exceed the device's storage capacity.

Open vial wastage occurs when vials are opened but not all the doses are completely consumed before expiration (e.g., only two doses used from a five-dose vial) as detailed by a previously published study and the WHO's Multi-Dose Vial Policy (MDVP)[4,11].

### Vaccine Specifications

Table 1 lists Niger's six EPI vaccines[12]^,^[13,14]. Information from previous studies was used to compute the cost of contaminated medical waste disposal[15]. The volumes of the diluents are considered, but only consume space at the IHC locations several hours prior to vaccine administration.

**Table 1:** Niger's EPI Vaccine Characteristics *Vaccines not included in current EPI schedule

Expanded Programme on Immunization (EPI) Vaccine	Immunization schedule	Doses per person	Doses per vial	**Packaged volume per dose (cm**^**3**^**)**	**Packaged volume per diluent (cm**^**3**^**)**	Route of administration	Preferred storage	Source
**Bacille Calmette-Guerin (BCG)**	Birth	1	20	1.2	0.7	Intra-dermal	Refrigerator	[11-13]
**Diptheria-tetanus-pertussis-hepatitis B-haemophilus influenza type B (DTP-HepB-Hib)**	6, 10, 14 weeks	3	1	16.8	None	Intra-muscular	Refrigerator	[11-13]
**Yellow Fever (YF)**	9 months	1	10	2.5	6.0	Sub-cutaneous	Refrigerator	[11-13]
**Oral Polio Virus (OPV)**	Birth, 6, 10, 14 weeks	4	20	1.0	None	Intra-muscular	Freezer	[11-13]
**Tetanus Toxoid (TT)**	1^st ^contact, 4 weeks, 6 months, 1 year	5	10	3.0	None	Intra-muscular	Refrigerator	[11-13]
**Measles (M)**	9 months	1	10	2.6	0.5	Sub-cutaneous	Refrigerator	[11-13]
Measles (M)*	9 months	1	5	5.2	0.5	Sub-cutaneous	Refrigerator	[11-13]
Measles (M)*	9 months	1	2	13.1	0.5	Sub-cutaneous	Refrigerator	[11-13]
Measles (M)*	9 months	1	1	26.1	0.5	Sub-cutaneous	Refrigerator	[11-13]

Niger currently supplies measles vaccines in 10-dose vials (2.61cm^3 ^per dose). Our analysis explores the effects of substituting the measles vaccine dose administered at age <1 year from the 10-dose vial with a 5, 2 or 1-dose vaccine presentation.

### Supply Chain Performance Metrics

Vaccine availability (percent of children arriving at a health centre who are able to receive their requested vaccine due to vaccine inventory availability) is computed for each simulation for each vaccine type at each IHC:

**Vaccine Availability **= (Number of patients receiving vaccine)÷(Number of patients arriving at an IHC) per year

The transport capacity utilization rate for each transport device (e.g., truck, cold box or vaccine carrier) and the storage utilization rate for each refrigerator and freezer are computed for each simulation run:

**Transport Capacity Utilization Rate **= Transport space consumed÷Total available transport space per shipment

**Storage Utilization Rate **= Storage space consumed÷Total available storage space per refrigerator or freezer

Using input values from Tables 1 and 2, the following equations are employed to calculate costs of using a single versus multi-dose vial (i.e., cost of vaccine dose administration, wastage, and disposal)[16]. A generally accepted discounted rate of 3% updated costs to 2011 United States dollars ($US)[17]:

**Cost of using single-dose vial **= Cost of administering a dose+Cost of disposal

**Cost of using multi-dose vial **= Cost of administering a dose+Cost of wasted doses+Cost of disposal

• ***Cost of administering a dose ***= (Cost of vaccine per dose*Number of doses administered)+(Cost of an administration syringe*Number of doses administered)+Cost of the total number of reconstitution syringes

• ***Cost of wasted doses ***= Cost of vaccine per dose*Number of wasted doses

• ***Cost of disposal ***= Safety box cost per dose+(Waste disposal cost per kg*(Weight of a vial+weight of a reconstitution syringe+weight of total administering syringes))

**Table 2:** Cost Inputs for Vaccine Variables

Variable	Mean	Min	Max	Source
**Cost per dose of vaccine ($US)**				
**Measles 10-dose**	0.246	-	-	[13,14]
**Measles 5-dose**	0.450	0.405	0.495	[16]
**Measles 1-dose**	0.943	-	-	[13,14]
**Volume per dose of vaccine (cm3)**				
**Measles 10-dose**	2.460	2.214	2.706	[13,14]
**Measles 5-dose**	5.220	4.698	5.742	[13,14]
**Measles 1-dose**	26.110	23.499	28.721	[13,14]
**Weight of vaccines and vaccine accessories (g)**				
**10-dose vial (empty)**	3.522	3.169	3.874	[16]
**5-dose vial (empty)**	2.517	2.265	2.768	[16]
**1-dose vial (empty)**	1.713	1.542	1.885	[16]
**Reconstitution syringe**	6.625	5.967	7.293	[16]
**Injection syringe**	6.625	5.967	7.293	[16]
**Cost of medical vaccine accessories (2009 $US) and waste disposal (2004 $US) adjusted to 2010 $US**				[17]
**Waste disposal cost per kg**	6.850	2.066	10.830	[15]
**Waste disposal cost per g**	0.0069	0.0021	0.0108	[15]
**Injection syringe**	0.070	-	-	[14]
**Reconstitution syringe**	0.060	-	-	[14]

The price per dose of the 2-dose measles vial size is unknown. The price per dose of the 5-dose vial is taken from a previous study which used linear regression analysis on all available vaccine vial sizes to estimate three price points[16]. All other vial size prices per dose are taken from the 2009 WHO Vaccine Volume Calculator[14].

Each vial size scenario is simulated and averaged over ten realizations. Due to stochasticity, the number of arriving patients in each scenario varies slightly across vial size scenarios.

#### Sensitivity Analyses

Sensitivity analyses systematically ranged the following parameters: inventory loss rate (range: 0-2%), shipping loss rate (range: 0-2%), storage capacity utilization (85-100%), population demand (static [i.e., number of patients in a month is fixed based on projected population estimates and does not fluctuate from month to month] versus dynamic monthly distribution [i.e., number of vaccine recipients in a given month draws from a Poisson distribution with a mean of (λ)]), and percentage of the target population that actually arrives at clinics to be vaccinated (60-100%).

Model validation consisted of running similar scenarios in a previously published deterministic equation-based model and observing convergence in results from both models (Lee BY, et al: Impact of introducing the pneumococcal and rotavirus vaccines into the routine immunization programme in Niger, submitted). We also compared the trends seen in our experiments with those from other similar field studies in other countries[18].

## Results

Sensitivity analyses demonstrated that varying the patient demand (static versus dynamic), shipping and inventory loss, and storage capacity utilization did not notably affect the pattern of results. Varying the target population that actually arrived at a clinic to be vaccinated had noteworthy effects on vaccine availability, transport and storage utilization. Therefore, the following results report from scenarios representing 1% inventory and shipping loss, 100% capacity utilization, and dynamic monthly demand for target population sizes of 100%, 80% and 60%.

### Overall Impact

Our model results suggest that the larger packaged volumes per dose of the 1-dose, 2-dose, and 5-dose measles vial sizes reduce vaccine availability at IHCs. Smaller vial sizes create greater bottlenecks in the already constrained region-to-district and district-to-IHC transportation devices. Moreover, while larger vial sizes result in more wasted doses, their lower price per dose provides relative cost savings. Additionally, the increased number and volume of vials and injection accessories (e.g., injection and reconstitution syringes) associated with smaller vial sizes results in substantial increases in waste disposal costs.

### Impact on Vaccine Administration

Switching from the 10-dose to the 5-dose, 2-dose and 1-dose measles vaccine vial size decreased the percent of arriving patients receiving vaccines (vaccine availability) from 90%, (58,482 of 583,575 measles vaccination opportunities being missed), to 87% (75,116 of 583,477 missed vaccination opportunities), 86% (84,307 of 583,722 missed measles vaccination opportunities) and 80% (117,167 of 583,264 missed measles vaccination opportunities), respectively, across the entire country.

Table 3 lists vaccine availabilities for other EPI vaccines across 60%, 80% and 100% target population sizes. Not only do the smaller vial size scenarios affect availability of the measles vaccine, but the average availability across other current EPI vaccines also decreased slightly following the switch.

**Table 3:** Vaccine Availability across Measles Vaccine Vial Size Scenarios and Target Population Sizes

Measles Vaccine Vial Size Scenario	10-doses per vial	5-doses per vial	2-doses per vial	1-doses per vial
**100% Target Population Size**				
Bacille Calmette-Guerin Tuberculosis (BCG)	79%	79%	78%	76%
Diptheria-tetanus-pertussis-hepatitis B-haemophilus influenza type B (DTP-HepB-Hib)	84%	84%	82%	80%
Measles	90%	87%	86%	80%
Oral polio (OPV)	80%	80%	78%	76%
Tetanus toxoid (TT)	84%	84%	83%	80%
Yellow fever (YF)	79%	79%	78%	76%
**80% Target Population Size**				
BCG	84%	85%	85%	84%
DTP-HepB-Hib	86%	86%	86%	84%
Measles	94%	92%	89%	84%
OPV	80%	80%	80%	79%
TT	86%	86%	86%	84%
YF	84%	85%	85%	84%
**60% Target Population Size**				
BCG	81%	81%	81%	80%
DTP-HepB-Hib	91%	91%	91%	89%
Measles	93%	91%	91%	90%
OPV	82%	82%	82%	82%
TT	90%	90%	90%	89%
YF	81%	81%	81%	80%

### Impact on Vaccine Transport

**Figure 2:**
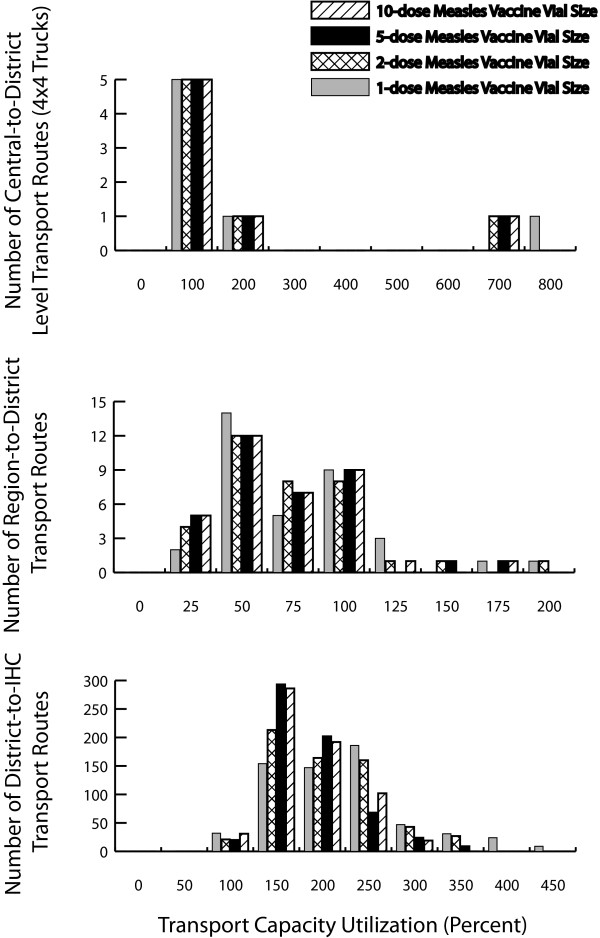
**Truck Capacity Utilization for Multi-dose and Single-dose Measles Vaccine Presentations**.

Figure 2 shows frequency histograms for transport capacity utilization between the region and district, and district and IHC levels across different vial size scenarios. In the first column, each bar in a histogram represents the number of transport vehicles along a route experiencing a certain percentage of capacity utilization. For example, in the first panel, for all vial size scenarios, 5 of the 7 central-to-region transport routes outside the two shipping loops are filled to 100% of their available capacity. In this figure, any transport capacity utilization exceeding 100% is the demand requested utilization from a downstream location, while the actual utilization of the vehicle is limited to 100%. For example, if a clinic needs 100 vials but their transport device can only carry 50, their transport device will be overfilled, its transport utilization will be 200%, but it can send at most 100% (or 50 of 100 vials). Those portions of the orders above 100% go un-served and are added to the next shipment.

There was enough cold transport capacity for vaccine shipments from the central store to the regional stores in the two shipping loops for all vial size scenarios only changing the median utilization from 66% for the two cold trucks which distribute vaccines to 7 regional stores, to 66%, 71% and 77% for the 5-dose, 2-dose and 1-dose vial sizes, respectively. However, the 7 district stores in the region that procure their own vaccines in 4 × 4 trucks consistently experienced overfill from a median across transport routes of 68% transport capacity utilization (range: 32-615%) for the 10-dose vial size scenario, to 74% (range: 32-616%), 80% (range: 32-659%) and 90% (range: 32-720%) for the 5-dose, 2-dose and 1-dose vial size scenario, respectively, resulting in many necessary vaccines in excess of available transport capacity not being delivered.

Similarly, the bottleneck in transport continues from regional to district level stores, wherein with the exception of one district store that has no cold storage capacity, the median 4 × 4 truck capacity utilization across transport routes changed from 53% (4-164%) in the 10-dose vial size scenario to 55% (range: 4-164%), 56% (range: 5-175%) and 56% (5-192%), respectively.

Bottlenecks in transport continue to have an impact from district to IHCs wherein the median vaccine carrier capacity utilization across transport routes changed from 149% (range: 83-300%) in the 10-dose vial size scenario to 154% (range: 82-322%), 168% (range: 78-344%) and 189% (range: 84-402%) for the 5-dose, 2-dose and 1-dose vial size scenarios, respectively. Reducing the target population to 80% and 60% reduces the median utilizations of cold trucks by up to 20% from the previous scenario, 4 × 4 trucks (central to regional) by up to 18%, 4 × 4 trucks (region to district) by up to 10%, and vaccine carriers by up to 20%, respectively.

### Impact on Vaccine Storage

**Figure 3:**
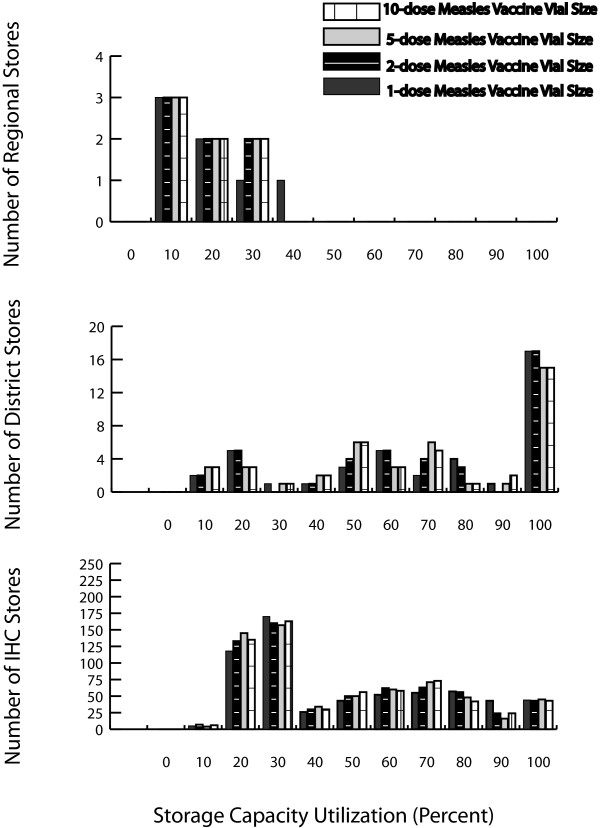
**Storage Capacity Utilization for Multi-dose and Single-dose Measles Vaccine Presentations**.

Figure 3 shows frequency histograms of the storage capacity utilization at the regional, district, and IHC stores across vial size scenarios. While cold room capacity utilization at the central level did increase for some scenarios from 65% in the 10-dose vial size scenario to 65%, 70% and 76%, for the 5-dose, 2-dose and 1-dose vial size scenarios, respectively, there was ample room for future vaccine introductions or storage of other temperature sensitive products.

The regional level also had enough capacity to accommodate the added volume from the smaller vial sizes, only changing the median capacity utilization for some scenarios across regional stores from 12% (range: 1-27%) in the 10-dose vial size scenario to 15% (range: 1-27%), 16% (range: 1-29%), and 17% (range: 1-33%) in the 5-dose, 2-dose, and 1-dose vial size scenarios, respectively.

Storage capacity bottlenecks first emerged at the district level with median district level refrigerator utilization across district stores changing slightly for some scenarios from 63% in the 10-dose vial size scenario to 63%, 67%, and 73% (range for all scenarios: 3-100%). The number of district stores exceeding 80% of their available refrigerator capacity changed for some scenarios from 17 to 16, 17 and 18 stores out of 42, many of which exceeded even 95% of their available storage space for the 5-dose, 2-dose and 1-dose vial sizes, respectively.

Given the persistent district level storage and transport bottlenecks with decreasing vial size, fewer vaccines were able to reach the IHC level and the median storage capacity utilization across IHCs did not change substantially from 33% (range: 3-99%) for the 5-dose vial size, 34% (range: 0-98%) for the 2-dose vial size, and 36% (range: 0-97%) for the 1-dose vial size compared to 34% (range: 4-98%) in the 10-dose vial size scenario. Moreover, the number of IHC stores exceeding 80% of their storage capacity decreased slightly in some scenarios from 67 to 61, 67 and 87 of 695 stores for the 5-dose, 2-dose and 1-dose vial sizes, respectively. Reducing the target population from 100% to 80% reduces the median storage capacity utilization across levels by 0-4% and from 80% to 60% by 5-20%.

### Impact on Vaccine Supply Chain Costs

Despite reductions in open vial waste with the 5-dose, 2-dose and 1-dose vial sizes compared to the 10-dose vial size, the number of doses saved was outweighed by the costs associated with the increasing price per dose and cost of waste disposal as vial sizes decreased. The number of wasted doses of the measles vaccine decreased from 1,279,450 for the 10-dose vial size to 442,398, 96,357 and 0 for the 5-dose, 2-dose and 1-dose vial sizes, respectively. However, the total costs of wasted doses from open vial waste increased from $33,391US for the 10-dose vials ($0.25US/dose) to $42,205US (range: $38,046US-$46,894US) for the 5-dose vials ($0.48US/dose, range: $0.43US-$0.53US) per year across Niger on account of the increasing cost per dose with decreasing vial size. The cost of the 2-dose vial was unknown and the 1-dose vial size ($0.94US/dose) produced no open vial waste. Similarly, as the vial size decreased, the cost of vaccine administration more than doubled from $179,779US in the 10-dose vial size scenario to $287,131US (range: $263,194US-$314,124US) and more than tripled to $586,214US in the 5-dose and 1-dose vial sizes, respectively. Finally, the increased volume of vials and injection material per dose with smaller vial sizes resulted in increased costs of contaminated waste disposal from $31,588US to $33,275US and $55,025US. All three cost components combined translate to a total cost increase from $244,759US to $362,611US ($334,515US-$403,279US) and $586,214US, or to an increase in the cost per injection from $0.47US in the 10-dose vial size scenario to $0.71US ($0.66US-$0.77US) and $1.26US in the 5-dose and 1-dose vial size scenarios.

Given that there were fewer administered doses with the 5 and 1-dose vial size scenarios, these costs would likely increase if there were enough storage and transport capacity to achieve baseline measles vaccine supply ratios or higher.

## Discussion

Results suggest that while selecting smaller vial sizes substantially reduces open vial waste, current storage and transport resources at the district and IHC levels of the Niger vaccine supply chain are pushed to their capacity, which results in lower vaccine availability of smaller vial sizes at the IHC level. The increased space requirement associated with the smaller vial sizes could also limit the supply chain's flexibility to respond to sudden changes that would require extra capacity (e.g., vehicle breakdown, refrigerator failure, etc.) or future anticipated increases in vaccine throughput from the introduction of new and under-utilized vaccines. Moreover, decreasing the vial size would increase the cost of the vaccine per dose and the total volume of vials and safe injection equipment in the supply chain, thereby increasing the costs of vaccine administration and waste disposal, which far outweigh the costs saved from the decrease in wasted doses.

Several noteworthy effects resulted when we reduced the target population to 80% and 60% of total EPI-eligible children: 1) smaller session sizes were associated with higher vaccine wastage, and 2) the wastage rate varied across vaccine types (i.e., liquid versus lyophilized), and across vial sizes (i.e., 1, 5, 10 and 20 doses per vial). For example, OPV, DTP-HepB-Hib, and TT had lower wastage than measles, BCG and YF because according to the WHO's MDVP, these liquid vaccines can be kept up to 4 weeks after opening[11]. The smaller vial sizes experienced higher wastage than the larger vial sizes, particularly for smaller session sizes. These are important findings that have also been reported in other field programme evaluations in similar settings, and suggest tailoring vial size selections to anticipated session sizes[18]. For example, 1 and 2-dose vials can be used efficiently for birth doses, whereas 10 and 20-dose vials would be better suited for mass vaccination campaigns or clinics with larger volumes of patients. This may require customizing re-order policies for specific regions, districts, or clinics where demand for vaccines is highly variable. Scaling up orders based on global estimates of vaccine-specific open vial waste for the whole supply chain, especially ones with high population variability may lead to over or under-ordering vaccines in some locations.

Our findings highlight the importance of considering the effects on existing supply chain resources when changing the presentations of vaccines in a country's EPI. Introducing a new vaccine presentation into a supply chain with limited resources can not only affect the supply of that vaccine, but also of all other vaccines in the EPI, which could ultimately hinder important infectious disease control efforts. Simulations of vaccine distribution through supply chains prior to any change in the EPI vaccines or to the structure of the supply chain itself can help pinpoint series of locations along the chain where bottlenecks, stock-outs, or overstocking may occur. These evaluations can then inform public health decision makers and logisticians on where the addition of new resources (e.g., refrigerators, freezers, trucks, etc.) would be most beneficial.

Additionally, our findings also illustrate the importance of considering medical waste when introducing a new vaccine presentation or vaccine technology. Improperly disposing of waste can result in harmful exposure to blood-borne pathogens. The costs of effective systems to safely remove contaminated medical waste and prevent infection often include costs for separating, sorting, treating, and transporting different kinds of waste products to disposal facilities where they can be either buried or incinerated[19].

However, rather than discourage the use of single-dose vaccine presentations, our findings simply help identify some of the programmatic and economic repercussions of switching from 10-dose to 5-dose, 2-dose or 1-dose vaccine presentations. Smaller vial size presentations offer certain benefits not accounted for by our study. The 1-dose presentation may allow for more consistent dose-size administration, reduce the risk of cross-contamination from repeated entry by reconstitution syringes and injection syringes for administrations, and provide more convenience to health care workers who would otherwise have to keep track of the number and volume of doses they withdraw. Eliminating open vial wastage may also alleviate the need for policies to plan when and when not to open new vials in response to arriving patients in order to minimize open vial wastage.

Our study illustrates how models can be applied in different scenarios to identify the effects of decisions not immediately apparent. Models have been commonly used for decision making in many other industries, such as manufacturing[20], meteorology[21], transportation[22], aerospace[23], finance[24], and sports and rehabilitation[25]. Conversely, their uses in public health have been relatively less extensive[26-28]. Of late, models in public health have assisted responses to the spread of infectious disease (e.g., the 2009 H1N1 influenza pandemic) and health-care associated infections[29-33], but much of their potential applications remain untapped.

### Limitations

Given that models are by definition, simplified representations of real life, they cannot capture every potential factor, event, or outcome[34,35]. Additionally, the data parameters included in our model are collected up to 2010 and may not represent future changes that may occur in the Niger vaccine supply chain. Due to the paucity of available data, the actual daily patient demand may vary from our estimated demand. Furthermore, developing our model involved substantial data collection including obtaining records from and conducting interviews at different locations in and out-of-country and came from a wide variety of sources. Thus, parameter values may vary in accuracy and reliability. Despite these limitations, sensitivity analyses demonstrated that model outcomes are robust under a wide variety of circumstances.

## Conclusions

While smaller vial sizes of the measles vaccine lead to decreases in open vial waste, the space utilization of several storage sites and transport routes in the Niger vaccine supply chain are insufficient to accommodate their higher volumes per dose, which result in lower vaccine availability for arriving patients. Moreover, at higher prices per dose, the costs of wasted doses and contaminated waste disposal ultimately outweigh the benefits provided by reducing or eliminating open vial waste.

## Competing interests

The authors declare that they have no competing interests.

## Authors' contributions

TA, STB, AD, BAN, JR, JSW, SC, RRB, SK, HK, DLC, ARW, AJ, SRW, WAV, DSB, BYL have read, approved, and contributed significantly to the work.

## Pre-publication history

The pre-publication history for this paper can be accessed here:

http://www.biomedcentral.com/1471-2458/11/425/prepub
